# Long COVID in adults – a current review of the long-term health effects following SARS-CoV-2 infection

**DOI:** 10.25646/13622

**Published:** 2026-02-25

**Authors:** Julia Nübel, Ann-Kristin Beyer, Lisa Kümpel, Grit Eckert, Dinara Yessimova, Katharina Heldt, Agata Mikolajewska, Giselle Sarganas

**Affiliations:** 1 Robert Koch Institute, Department of Epidemiology and Health Monitoring, Berlin, Germany; 2 Robert Koch Institute, Method Development, Research Infrastructure and Information Technology (MFI), Berlin, Germany; 3 Robert Koch Institute, Centre for Biological Threats and Special Pathogens (ZBS), Berlin, Germany

**Keywords:** Long COVID, Post COVID syndrome, Post COVID-19 condition, Post-acute sequelae of COVID-19, SARS-CoV-2 pandemic, Post-acute infection syndrome, COVID-19, SARS-CoV-2, Public health

## Abstract

**Background:**

Long-term health effects associated with SARS-CoV-2 pose major challenges for public health and health research worldwide.

**Methods:**

Based on an ongoing literature review, a narrative review (as of June 2025) on the epidemiology and public health implications of long COVID in adults was compiled.

**Results:**

According to population-based, controlled studies, long COVID symptoms occur with a frequency of approximately 10 to 15 % in adults infected with SARS-CoV-2. In addition to COVID-19 vaccination status and virus variant, the risk of experiencing long COVID symptoms is primarily influenced by pre-existing health conditions and sociodemographic factors. In most affected individuals, long COVID symptoms resolve within a year. Particularly multiple and prolonged symptoms can be associated with significant impairments in quality of life, everyday functioning and social participation, as well as an increased need for healthcare. In addition, there is growing evidence of an infection-associated increase in newly diagnosed symptom complexes, organ damage and chronic diseases, contributing to the ongoing public health relevance of long COVID.

**Conclusions:**

Long COVID is not only a major burden for those affected and their families, but also has unpredictable long-term consequences for public health and the healthcare system.

## 1. Introduction

Since summer 2020, reports have been circulating, initially on social media [1] and then increasingly in international clinical and epidemiological studies, of health complaints that persisted beyond the acute phase of SARS-CoV-2 infection, recurred or even developed anew – even in individuals who initially had mild or no COVID-19 symptoms and were previously healthy. In 2021, this novel infection-associated condition was defined as ‘post COVID-19 condition’ by the World Health Organization (WHO) and included in the International Statistical Classification of Diseases and Related Health Problems (ICD-10) [2–4]. The long-term health effects of SARS-CoV-2 infection are generally referred to as ‘long COVID’ and, according to current knowledge, include non-specific complaints and symptom complexes that cannot be explained otherwise, as well as the worsening of pre-existing health conditions, organ damage and increased incidence of certain chronic diseases [5–8].

**Figure 1:**
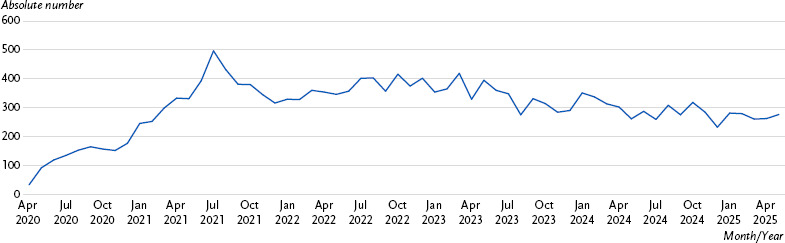
Monthly publications on long COVID (references indexed in PubMed). Source: LitCovid [9].

A significantly delayed recovery or lasting impairments to health, quality of life, and daily functioning impose a substantial burden on affected individuals, their families, and society at large – beyond the acute consequences of COVID-19. Long COVID therefore remains the subject of intensive research (Figure 1). Nevertheless, overall assessments from a public health perspective remain challenging, partly due to the diversity and complexity of the clinical picture, the different pathomechanisms and unclear diagnostic criteria. This narrative review summarises current knowledge regarding the definition, epidemiology, and consequences of long COVID in adults, as well as briefly describes the healthcare provided to patients with long COVID in Germany.

**Key messages**

► In addition to non-specific symptoms (e.g. fatigue, cognitive dysfunction and respiratory complaints), long COVID also includes organ damage, worsening of pre-existing health conditions and incident medical diagnoses following SARS-CoV-2 infection.
► Long COVID symptoms occur in approximately 10 to 15 % of adults in the period of at least twelve weeks after SARS-CoV-2 infection.
► Approximately one in four to five people with long COVID symptoms report significant limitations in performing daily activities due to their symptoms.
► The risk of experiencing long COVID symptoms is higher in women, people with pre-existing health conditions and unvaccinated individuals, among others.
► An estimated 15 % of those affected by long COVID still have symptoms after one year.

## 2. Methods

### 2.1 Literature review

This work is based on continuous literature searches, considering research literature on long COVID from January 2020 to June 2025. To initiate a literature screening workflow, a search string alert setup was implemented in the PubMed database in July 2021 for a weekly literature search on long COVID. First, the introduced Supplementary Concept ‘postacute COVID-19 syndrome’ [Supplementary Concept] was thematically combined with other keyword combinations related to long COVID using the Boolean operator OR, followed later by the MeSH term ‘post-acute COVID-19 syndrome’ [MeSH]. The keyword combinations used were combined from individual elements relating to chronicity (e.g. persist * OR long OR post etc.) and Covid (Covid * OR Sars-Cov-2) using the Boolean operator AND. After the introduction of the LitCovid Long Covid filter LitCLONGCOVID [filter] in PubMed, this was set up as an additional alert in July 2022. Between 2021 and 2025, the long COVID team at the Robert Koch Institute (RKI) made several updates or additions to the search strings in response to changes in the research literature on long COVID. Due to the large number of references, filters were integrated for individual publication types (e.g. for meta-analyses and reviews) and temporary, additional search strings focusing on individual aspects (e.g. COVID-19 vaccination, virus variants, and incident diseases resulting from SARS-CoV-2 infection) were added in order to address specific research questions in addition to the broad literature search on long COVID. In addition to searching PubMed, Cochrane CENTRAL and the preprint search engine ‘PreVIEW’ were regularly searched, as well as the websites of PROSPE-RO (international prospective register of systematic reviews) and OSF (Open Science Framework). Further manual searches were conducted with regard to publications of the long COVID Surveillance by the British Office for National Statistics (ONS) and the US Centers for Disease Control and Prevention (CDC). In addition, press releases and guideline publications in Germany were regularly checked as well as secondary data analyses on long COVID from statutory social insurances, such as health insurance reports and publications by the Central Research Institute for Ambulatory Health Care in Germany (Zi). The last automated PubMed search took place on 30 June 2025. During the entire search period, a total of 4,782 references were transferred to an Endnote database after an initial title/abstract screening.

### 2.2 Literature selection

The literature selection was carried out by the long COVID team at the RKI, based on ongoing screening of newly published articles in English or German focusing on the epidemiology and public health relevance of long COVID. To describe the international findings, this article primarily includes current publications with a higher level of evidence (umbrella reviews, meta-analyses and systematic reviews). In the case of original studies, particular consideration was given to prospective cohort studies and population-based studies with a control group (controlled studies) that compared individuals with and without a history of SARS-CoV-2 infection in terms of existing health complaints and symptoms. The focus was on studies that were based on the WHO’s time criterion for defining a ‘post COVID-19 condition’ and examined health complaints in the period of at least three months after infection. In addition, population-based studies that examined long COVID in predominantly non-hospitalised individuals and controlled for possible influencing factors (e.g. pre-existing conditions and diseases) were favoured to describe the epidemiological data.

## 3. Terminology and definition

The term ‘long COVID’ was coined by those affected in the early stages of the SARS-CoV-2 pandemic [1]. However, there are inconsistent, sometimes country-specific terminologies and definitions for the long-term health effects of SARS-CoV-2 infection [5, 6, 10, 11]. What all terms have in common is that they have a temporal basis for definition, but do not explicitly select the symptoms and diseases to be considered.

**Figure 2:**
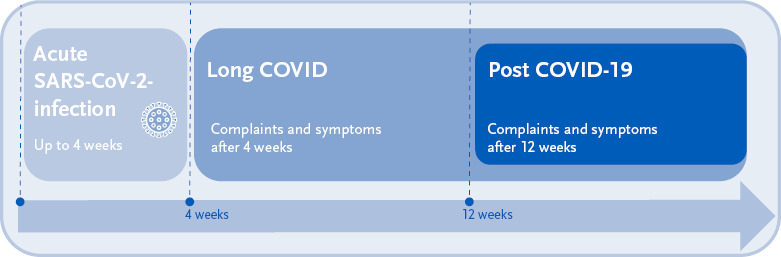
Temporal classification of long-term health effects following SARS-CoV-2 infection.

Based on the guidelines published at the end of 2020 by the British National Institute for Health and Care Excellence (NICE) on the care of patients with long-term effects of COVID-19, the term ‘long COVID’ is commonly used to describe health problems from four weeks or more after an acute SARS-CoV-2 infection (Figure 2) [12]. The terms ‘post-acute sequelae of COVID-19’ (PASC) and ‘post-acute COVID-19 syndrome’ (PACS) are also used as synonyms for long COVID [13, 14]. The so-called ‘post COVID-19 syndrome’ (PCS) encompasses symptoms that are still present more than twelve weeks after a SARS-CoV-2 infection [12, 13, 15]. This temporal-descriptive approach is also followed by the medical S1 guideline ‘Long/Post-COVID’ of the Association of Scientific Medical Societies in Germany (AWMF) in its definition of the terms [16].

Based on an international and scientifically based Delphi consensus process, the WHO published a clinical case definition of a ‘post COVID-19 condition’ (PCC) in October 2021 [2], which has since been used or adapted primarily in scientific studies [6, 10, 17]. According to the WHO definition, ‘post COVID-19 condition’ encompasses health complaints that usually persist or newly develop three months after a confirmed or probable SARS-CoV-2 infection and cannot be explained by any other diagnosis. This includes symptoms that last for at least two months or relapse and fluctuate, and which generally have a negative impact on everyday functioning. A separate case definition of a post COVID-19 condition for children and adolescents was added by the WHO in February 2023 [18]. According to the WHO, both case definitions are to be considered provisional and may need to be adapted to new research findings.

In July 2024, the US National Academies of Sciences, Engineering, and Medicine (NASEM) published a working definition based on new scientific evidence that had become available in the meantime [19]. For the first time, this definition moves beyond a symptom-based classification of ‘long COVID’. Instead, it comprehensively characterises the condition as an infection-associated chronic condition affecting at least one organ system. To meet this definition, the condition must have been present for at least three months, whether continuous, relapsing, remitting, or progressive, and may result in impaired daily functioning. In addition to individual or multiple symptoms, the NASEM definition explicitly considers diagnosable symptom complexes and diseases, such as newly developed medical conditions or an exacerbation of a pre-existing health condition. Furthermore, according to this definition, long COVID can also follow asymptomatic or unrecognised SARS-CoV-2 infection.

Accordingly, existing definitions of long COVID differ primarily in terms of the time interval since the previous SARS-CoV-2 infection (e.g. ≥ four vs. ≥ twelve weeks) and with regard to the consideration of diagnosable symptom complexes and diseases. However, in clinical practice, a narrower understanding of long COVID is favoured, in accordance with the WHO definition, namely as a term for novel, persistent, or recurrent symptoms following SARS-CoV-2 in-fection that are not explained by an alternative diagnosis [6, 15]. For the diagnosis of PCS, the German S1 guideline considers not only persistent symptoms and post-acute new-onset sequelae attributed to SARS-CoV-2, but also the COVID-19-related exacerbation of pre-existing conditions and symptoms resulting from intensive care treatment (post-intensive care syndrome (PICS)) [16].

In this article, ‘long COVID’ is used as an umbrella term to encompass the entire period beyond the acute SARS-CoV-2 infection, as well as the full range of possible long-term health effects. However, consistent with WHO, NASEM, and German S1 guidelines, the primary focus is on health complaints occurring at least three months following SARS-CoV-2 infection (PCS). Accordingly, the presentation of findings will explicitly state whether referenced studies met this temporal criterion. The term ‘post COVID-19 condition’ is only used for medically diagnosed cases (according to the ICD) in the description of healthcare provision and administrative statistics.

## 4. Clinical picture

Long COVID encompasses a variety of possible long-term health effects following SARS-CoV-2 infection that can affect different organ systems and occur individually or in combination [5–7]. Since the symptoms vary considerably in their clinical manifestation and course, it has not yet been possible to define a uniform clinical picture for long COVID. The underlying causes and mechanisms of the disease are also not yet fully understood. For example, there is evidence that the SARS-CoV-2 virus remains in the body (known as ‘virus persistence’) and thus leads to health problems despite the acute infection subsiding. Another hypothesis suggests that long COVID is caused by the reactivation of latent viruses (e.g. Epstein-Barr virus) as a result of SARS-CoV-2 infection. Furthermore, the SARS-CoV-2 virus may cause misdirected immunological processes that target the body’s own cells or tissues and lead to chronic inflammation and blockages of small blood vessels (known as ‘endothelial dysfunction’). Changes in the gut microbiota may also be involved in the development of long COVID [6, 8].

### 4.1 Long COVID symptoms

Non-specific health complaints following SARS-CoV-2 infection (known as ‘long COVID symptoms’) include, in particular severe exhaustion/tiredness (known as ‘fatigue’) and cognitive impairments such as concentration and memory problems (known as ‘brain fog’), but also lasting respiratory symptoms such as shortness of breath and persistent cough [20–23]. Similarly, the WHO’s clinical case definition of a post COVID-19 condition also lists fatigue, shortness of breath and cognitive dysfunction as symptoms frequently associated with long COVID [2]. In addition to these three symptom complexes, meta-analyses of controlled studies identified a significantly increased risk of numerous other post-acute complaints compared to individuals without confirmed infection, such as changes in smell and taste, hair loss, palpitations, chest pain, muscle or joint pain, and gastrointestinal complaints [24, 25].

There was also an increased risk of ‘post-exertional malaise’ (PEM) [24], which was also identified as a ‘core outcome’ of long COVID in adults based on an international Delphi consensus process [21]. PEM (synonymous with ‘crash’) refers to a pronounced worsening of symptoms that can occur even after mild physical or mental exertion, usually lasts until the following day and can persist for several days or weeks [26].

### 4.2 Myalgic encephalomyelitis/chronic fatigue syndrome (ME/CFS)

In some patients with long COVID, myalgic encephalomyelitis/chronic fatigue syndrome (ME/CFS) can be identified as a particularly severe subtype [27]. ME/CFS is characterised by PEM as the cardinal symptom and is associated with significant limitations in quality of life, physical and mental functioning in everyday life, and social participation [28–31].

It is currently unclear how many people are affected by ME/CFS after a SARS-CoV-2 infection. Analyses of secondary data from statutory health insurance (SHI) companies in Germany show an approximately threefold higher risk of ME/CFS after COVID-19 disease compared to non-infected individuals [32]. Accordingly, following the SARS-CoV-2 pandemic, ongoing waves of COVID-19 are expected to lead to an increase in ME/CFS cases in the population [28, 33].

### 4.3 Further long-term health effects

Epidemiological and clinical observational studies have reported further possible symptom complexes (e.g. postural orthostatic tachycardia syndrome (POTS)), organ damage (e.g. chronic lung damage), worsening of pre-existing health conditions, and increased incidence of new medical diagnoses (including certain chronic, non-communicable diseases) in association with a previous SARS-CoV-2 infection [5–8]. These include neurological manifestations and neurodegenerative diseases (e.g. dementia), metabolic diseases (e.g. diabetes mellitus), cardiovascular diseases and autoimmune diseases, as well as gastrointestinal disorders. Accordingly, a meta-analysis of controlled studies also demonstrated a higher incidence of numerous medical diagnoses at least twelve weeks after SARS-CoV-2 infection compared to the non-infected control group [7]. Increased incidence rates are particularly evident in the first year after SARS-CoV-2 infection, but have also been reported for single organ systems in longer observation periods of up to three years after infection [34–37].

A population-based cohort study from Germany (Corona Monitoring Local (CoMoLo)) also demonstrated that, following a period of over a year since previous SARS-CoV-2 infection, there was an increased risk of a variety of health complaints compared to non-infected control subjects, including new conditions of the lungs, liver, kidney, as well as cardiovascular or metabolic diseases [38]. Similarly, analyses of German SHI data showed that adults had significantly more new-onset morbidity at least three months after a documented COVID-19 disease than controls without a confirmed SARS-CoV-2 infection [32]. Increased incidences were reported, for example, for autoimmune diseases [39] and atopic dermatitis (neurodermatitis) [40].

### 4.4 Comparison with other post-acute infection syndromes

Long-term health effects (including ME/CFS) have already been observed after other viral infections, such as influenza or Pfeiffer’s glandular fever caused by the Epstein-Barr virus (known as ‘post-acute infection syndromes’ (PAIS)) [41]. Current study results suggest that in both COVID-19 and seasonal influenza, the burden of post-acute health problems may actually be higher than during the acute phase of the illness [42]. In COVID-19, however, both the acute and postacute phases of the disease showed a greater impact on all organ systems (with the exception of the respiratory tract) compared to influenza. Other studies also point to a higher risk of long-term health effects after SARS-CoV-2 infection than after influenza infection [34, 43–49], but the findings are heterogeneous [50, 51]. In addition, there are only a few studies to date that have investigated long COVID in comparison with non-SARS-CoV-2-associated post-acute infection syndromes, so further research is needed.

## 5. Epidemiological data

The majority of international studies on the epidemiology of long COVID refer to symptoms that cannot be explained by other causes, based on the WHO case definition [2]. The full range of possible long-term health effects associated with SARS-CoV-2 infection, including incident medical diagnoses or the worsening of a pre-existing condition, cannot yet be estimated and is not considered in the presentation of the epidemiological data. Furthermore, when interpreting the findings, it should be noted that severely affected individuals (e.g. those with ME/CFS) may generally underrepresented in population-based studies.

### 5.1 Frequency

Prevalence estimates for long COVID vary considerably between individual studies and are not directly comparable due to the large methodological heterogeneity [52]. Differences arise, among other things, depending on the data basis, composition of the study population, long COVID definition, duration of follow-up and the consideration of possible influencing factors [10, 17, 22, 52, 53]. Furthermore, the majority of studies on long COVID do not have a control group and focus solely on non-specific symptoms that are generally common in the general population, e.g. in the context of other (possibly pre-existing) conditions. Consequently, the symptoms reported cannot be clearly attributed to a prior SARS-CoV-2 infection [17, 22, 52, 53]. As a result, some previous systematic reviews and meta-analyses have reported very high prevalence estimates of long COVID symptoms following SARS-CoV-2 infection [52]. For example, a metaanalysis of prospective studies estimates the frequency of at least one persistent or new symptom from three to six months after a confirmed COVID-19 diagnosis to be over 50 % [22].

More reliable estimates come from controlled studies that have compared the occurrence of health complaints in people with and without a history of SARS-CoV-2 infection. These studies indicate a comparatively low prevalence of long COVID [52, 54]. For example, a meta-analysis of controlled studies conducted up to mid-February 2023 estimated the pooled prevalence of at least one reported long COVID-associated symptom at least three months after confirmed SARS-CoV-2 infection to be 40.9 %. Among participants in the control group (without confirmed infection), the frequency was 25.4 %. The prevalence difference between the infection and control group was therefore 15.5 %, meaning that the reported symptoms among participants in the infection group can only be ascribed to a prior SARS-CoV-2 infection in 15.5 % of cases [24]. Based on a meta-analysis of controlled studies conducted up to November 2021, the pooled risk difference for long COVID symptoms between SARS-CoV-2-infected individuals and the control group was estimated at 10.1 % [53]. This indicates that, at twelve weeks post-infection, symptoms that were related to the preceding SARS-CoV-2 infection occurred in approximately one in ten infected individuals. In summary, based on meta-analyses of controlled studies, it can be assumed that long COVID symptoms occur in adults at a frequency of approximately 10 to 15 % in the period of at least twelve weeks after SARS-CoV-2 infection. However, both meta-analyses also included studies with non-representative, specific study populations (such as only severe COVID-19 cases with hospitalisation). When all SARS-CoV-2-infected in the population are taken into account (including cases with asymptomatic or unnoticed SARS-CoV-2 infection), a lower frequency of long COVID symptoms must therefore be assumed [22, 25, 53].

When limitations of everyday functioning due to symptoms are also taken into account (as required in the WHO case definition [2]), a lower prevalence is to be expected, as only one in four to five people with self-reported long COVID symptoms report significant day-to-day activity limitation due to the symptoms [55, 56]. Accordingly, ten British cohort studies recorded frequencies of long COVID symptoms that limited day-to-day function (twelve weeks or more after SARS-CoV-2 infection) between 1.2 % and 4.8 % [57]. Other studies also suggest a prevalence of less than 10 % for long COVID symptoms after SARS-CoV-2 infection that are associated with functional limitations [58, 59]. Lower estimates are also seen when adjusting [24], for example for pre-existing health complaints and diseases [60, 61]. For instance, the global risk of long COVID symptoms in adults three months after symptomatic SARS-CoV-2 infection, taking into account pre-existing health problems and the general presence of symptoms in the population, was estimated at 6.2 % overall, based on a multicentre, pooled analysis of population-based cohort studies from 22 countries [20].

Over time, there appears to be a decline in the incidence of long COVID-associated symptoms in adults with prior SARS-CoV-2 infection. A systematic review conducted at the end of 2022 already showed that the frequency of long COVID symptoms could vary depending on the virus variant [62]. Since then, there has been further evidence that the occurrence of long COVID symptoms is less common in infections with later SARS-CoV-2 variants such as Omicron and its sub-variants compared to earlier variants [63–65]. According to the RECOVER initiative of the US National Institutes of Health, the prevalence of long COVID-associated symptoms in infected individuals with the Omicron variant was only around five percentage points higher (10 % vs. 4.6 %) compared to non-infected individuals six months following infection [66]. However, recent studies suggest that, in addition to possible differences in the pathogenicity of the variants, other factors may be relevant to the decreasing risk of long COVID over time, such as higher immunity in the population due to COVID-19 vaccinations [66–72]. Further research is therefore needed to conclusively assess temporal trends, considering the interplay of virus variant, (re)infections and vaccination status [5, 52].

### 5.2 Risk and protective factors

Systematic reviews and meta-analyses consistently show that the risk of developing long COVID symptoms is particularly increased in cases of severe acute COVID-19 and hospitalisation, as well as in cases of prolonged hospitalisation due to COVID-19 [25, 52, 73–75]. However, since the majority of people infected with SARS-CoV-2 have a mild course of the disease, they account for more than 90 % of long COVID cases [5].

According to current findings, both the severity of acute COVID-19 disease and the risk of long COVID are strongly influenced by individual pre-existing health conditions [52, 53, 75]. Thus, an increased risk of long COVID symptoms has been reported for a number of pre-existing conditions and diseases, such as asthma and chronic obstructive pulmonary disease (COPD) [76], as well as cardiovascular disease [77]. There is also evidence that lifestyle factors such as smoking or physical inactivity may increase the risk of long COVID [75, 78].

Furthermore, the risk of long COVID symptoms depends on sociodemographic factors. Studies show that women are at higher risk of long COVID symptoms than men [20, 52, 74]. Gender differences are also reported in terms of the type, severity and duration of long COVID symptoms [25, 79], as well as the potentially underlying disease mechanisms. Gender-specific differences in the immune system and autoimmune responses are being discussed as a possible explanation for differences in long COVID risk between men and women [80, 81]. With regard to age, systematic reviews and meta-analyses suggest a higher risk of long COVID symptoms in middle-aged and older adults [6, 52, 53, 73, 75], although the findings are heterogeneous [52]. There are differences depending on the predominant symptoms, with brain fog or fatigue, for example, seeming to be more common among younger adults [82]. Furthermore, there is evidence that the risk of long COVID is influenced by social determinants [83]. An increased risk of long COVID has been observed in people living in areas with greater socioeconomic deprivation [84]. Overall, however, the data on the influence of social status or socio-spatial deprivation is limited and inconclusive [83]. Nevertheless, it can be assumed that existing social differences in pre-existing conditions, lifestyle factors and vaccination status contribute to social inequality in long COVID [85].

With regard to prevention, current systematic reviews and meta-analyses indicate that COVID-19 vaccination before initial SARS-CoV-2 infection offers a certain degree of protection against long COVID [52, 86–88]. Being fully vaccinated with at least two doses was shown to be more effective than a single vaccination. Overall, however, the findings are heterogeneous [87]. Furthermore, there is also evidence that antiviral drug therapy during acute SARS-CoV-2 infection could reduce the risk of long COVID, especially in older people and in patients at increased risk of severe COVID-19 [89, 90]. In addition, based on current knowledge, the best protection against long COVID is to avoid SARS-CoV-2 infection and reinfection [5], since individual studies suggest that each new SARS-CoV-2 infection is a risk factor for long COVID [66, 91]. However, due to the limited and heterogeneous findings, the role of reinfections with regard to the risk of long COVID has not yet been conclusively clarified [68, 92].

### 5.3 Duration

Systematic reviews and meta-analyses suggest that long COVID symptoms subside or disappear within a year in the majority of those affected [24, 73]. A comparison of the pooled, adjusted frequency of long COVID symptoms at three vs. twelve months after symptomatic SARS-CoV-2 infection also showed a significant decline over time. However, in a total of 15.1 % of participants with symptoms after three months, the complaints were still present after one year [20]. Further studies with longer follow-up periods of up to two years suggest that the chance of long COVID symptoms subsiding decreases over time, so that the complaints could become increasingly chronic [93, 94].

A longer duration of long COVID symptoms is particularly reported in cases with comorbidity, severe COVID-19 and previous hospitalisation [95]. The average time for long COVID symptoms to resolve after mild COVID-19 was estimated at four months, whereas the average recovery time for people hospitalised due to COVID-19 was approximately nine months [20]. A review based primarily on data from hospitalised individuals showed that 20 % of those infected still had at least one long COVID symptom three years after SARS-CoV-2 infection [95]. Furthermore, there are differences depending on the type of symptoms, with fatigue and neuropsychiatric symptoms in particular appearing to have a longer duration compared to other complaints [59, 95–97]. In addition, studies suggest that the decline in symptoms may be faster with the Omicron variant than with previous SARS-CoV-2 variants [94].

Overall, the data on the duration of long COVID symptoms is still very limited and heterogeneous [5]. Some reviews and meta-analyses report higher prevalence estimates for long COVID symptoms over time. However, the significance of these estimates is limited by the methodological heterogeneity of the largely uncontrolled studies. Furthermore, it remains unclear to what extent single, persistent symptoms are also accompanied by impairments in everyday functioning.

## 6. Care in Germany

### 6.1 Diagnosis and treatment

The AWMF S1 guideline ‘Long/Post-COVID’, which was developed under the leadership of the German Society for Pneumology and Respiratory Medicine with the participation of 36 other professional societies, provides diagnostic and therapeutic guidance with consensus-based recommendations for basic diagnostics in patients with long COVID based on the current state of knowledge [16]. To date, there are no specific diagnostic markers (examination or laboratory results) for long COVID, so the clinical diagnosis of a post COVID-19 condition (ICD-10 code U09.9!) must be made on the basis of a targeted, comprehensive medical history and examination [15, 16]. Besides, the differential diagnosis to distinguish between pre-existing morbidity, indirect consequences of the pandemic and ‘post-intensive care syndrome’ following intensive medical treatment due to COVID-19 poses a major challenge [16, 98].

According to the S1 guideline, the treatment of a post COVID-19 condition is symptom-oriented, whereby the presence of PEM in particular must be taken into account when coordinating an individual treatment plan [16]. There is currently no specific drug treatment available. However, the expert group ‘Long COVID Off-Label Use’ convened by the German Federal Ministry of Health (BMG) at the Federal Institute for Drugs and Medical Devices (BfArM) is currently developing evidence-based recommendations on drugs that are actually approved for other indications but can be used in so-called ‘off-label use’ in the treatment of post COVID-19 condition [99]. For approved symptom-oriented drug therapy (‘in-label use’), a ‘therapy compass’ from the expert group already provides guidance on suitable active substances and groups of active ingredients [100]. In addition, rehabilitation is an important part of clinical healthcare, especially in more severe cases [16, 101–103]. The S2k medical guideline ‘COVID-19 and (early) rehabilitation’ contains practical recommendations for rehabilitation of long COVID, based on a structured consensus process involving experts [104]. A ‘key issues paper for medical rehabilitation in post COVID syndrome’ was also presented by the German Pension Insurance in collaboration with experts to supplement the medical guidelines and describes requirements for an interdisciplinary treatment strategy for more complex cases [105].

General practitioners are usually the primary contact for people with long COVID complaints. However, depending on individual needs, interdisciplinary care with close cooperation between general practitioners and other specialist care providers may be necessary, also including special outpatient clinics or post COVID centres (e.g. for those most severely affected) [15, 16, 103]. The process for the interdisciplinary, coordinated and structured care (so called healthcare pathways) of patients with a post COVID-19 condition or suspected long COVID was specified at the end of 2023 in the long COVID guideline of the Federal Joint Committee [106]. To implement the guideline, several new services for the reimbursement of outpatient care for post COVID-19 condition were included in the Uniform Assessment Standard, the basis for the German physician reimbursement system, at the beginning of 2025. Among other things, services and surcharges for people severely affected were also included [107]. In addition, a post COVID-19 diagnosis is recognised as a special care need when prescribing outpatient remedies [108].

### 6.2 Treatment prevalence

According to data from the Central Research Institute for Ambulatory Health Care in Germany (Zi), a total of 8.6 % of all laboratory-confirmed COVID-19 patients (including children and adolescents) treated by SHI-accredited physicians during the study period from October 2020 to September 2021 had a diagnosed post COVID-19 condition (ICD-10 code U09.9!) [109]. Referred to the total number of SARS-CoV-2 infections notified during the study period in accordance with the German Infection Protection Act, the overall proportion of post COVID cases was 7.6 %. In line with epidemiological and clinical observational studies, a post COVID-19 condition was diagnosed more frequently in patients with pre-existing somatic and mental illnesses, irrespective of the age of those affected [103]. Furthermore, the Zi data indicate that the majority of patients with a medically diagnosed post COVID-19 condition did not require long-term treatment, as less than one-fifth of those affected had to be treated for more than two quarters between January and September 2021 [110].

**Figure 3:**
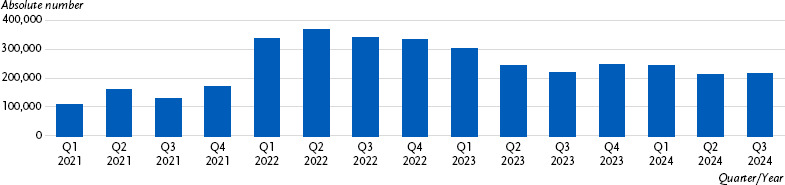
Number of patients diagnosed with post COVID-19 condition in Germany (statutory health insurance members with an ICD-10 code U09.9!). Source: Adapted from data provided by the Central Research Institute for Ambulatory Health Care in Germany (Zi) [111].

Over the course of the pandemic, the highest treatment prevalence was observed in the second quarter of 2022 with 371,705 cases (0.5 %, 50 per 10,000 SHI insured persons); since then, the numbers have been declining in accordance with the epidemiological data (Figure 3) [111].

Overall, the prevalence of post COVID-19 conditions observed in German SHI data is lower than the prevalence estimates for long COVID from international population-based studies with control groups. However, it can be assumed that existing long COVID symptoms are only recorded administratively in a proportion of those affected [44, 53, 112]. In addition to variations in diagnostic coding practices, the subjective need for treatment as well as healthcare utilisation behaviour are also relevant factors, particularly as these depend on symptom severity and the degree of functional impairment. A study from Western Australia showed that only 38.7 % of respondents with reported long COVID symptoms consulted a doctor two to three months after the acute infection due to related health problems (corresponding to 7.1 % of those infected) [113]. Moreover, existing symptoms may not always be interpreted as a consequence of SARS-CoV-2 infection by patients or their physicians – and therefore may be not documented as a post COVID-19 condition.

## 7. Individual and societal consequences

### 7.1 Quality of life, everyday functioning and social participation

Long COVID is associated with partly severe impairments in health-related quality of life and everyday functioning, e.g. in terms of cognitive performance [7, 22, 114, 115]. This is particularly true for those who are severely affected, such as those with multiple symptoms [23, 116, 117] or ME/CFS [28, 29]. Accordingly, analyses in OECD countries, taking into account the reduced quality of life, point to a significant loss of so-called ‘quality-adjusted life years’ (QALY) due to long COVID [118].

Functional limitations due to long COVID can in turn have a negative impact on the social participation of those affected, for instance in association with psychosocial challenges and changes [115] and with regard to work ability and occupational participation [119, 120]. A meta-analysis showed that three to six months after confirmed COVID-19, about onethird of those infected had not returned to work; after one year, the proportion was about 17 % [22].

According to data from the Scientific Institute of the German statutory health insurance company AOK, 1.8 % of employed people insured with the AOK in Germany were on sick leave at least once in 2023 due to the long-term effects of COVID-19 [121]. In addition to a diagnosed post COVID-19 condition, documented chronic fatigue syndrome and symptoms lasting longer than 28 days after acute COVID-19 were also considered. Compared to other illnesses, this resulted in relatively long periods of absence from work, averaging around 30 to 37 days per case. According to the German Social Accident Insurance (DGUV), occupational diseases related to COVID-19 had also been recognised for 362,757 people in Germany by December 2024 (as of 31 December 2024) [122]. In 2023, the German Pension Insurance approved around 1,499 invalidity pensions in association with post COVID [123].

Impairments of work ability, professional capacity or earning capacity due to long COVID also have societal and economic consequences. A report by the European Commission recorded a 0.3 to 0.5 % decline in the labour supply on the EU labour market in 2022 compared to the previous year associated with long COVID – particularly in terms of productivity losses, increased sick leaves, reduced working hours and increased unemployment [124]. Results from the OECD Patient-Reported Indicator Survey (PaRIS) suggest that sick leave or unemployment due to long COVID particularly affects people with pre-existing chronic conditions [11].

### 7.2 Health and use of healthcare services

People with long COVID report poorer self-rated mental and physical health [11, 114, 117, 125] and increased use of outpatient, inpatient and emergency medical services, accompanied by higher direct healthcare costs [126]. According to a US study from 2025, the current health economic burden of long COVID symptoms could already exceed that of other chronic diseases [127].

However, long-term health effects of SARS-CoV-2 infection may occur and result in healthcare utilisation even if they are not perceived as long COVID by those affected or not recognised and documented by doctors as a post COVID-19 condition (ICD-10 U09.9!). This may be particularly the case with regard to the exacerbation of pre-existing conditions or the onset of new diseases after a SARS-CoV-2 infection [5–7], as comprehensive SHI data analyses in Germany also suggest [32, 39, 40, 47]. Accordingly, reviews show that individuals who have recovered from COVID-19 still have increased use of health services and higher costs compared to non-infected individuals several months after acute SARS-CoV-2 infection [7, 126]. In addition, after three years, there was also an increased mortality rate among SARS-CoV-2-infected individuals, especially those with prior hospitalisation due to COVID-19 or neurological manifestations [37, 128]. Accordingly, SARS-CoV-2 infected individuals show a significant disease burden due to morbidity and mortality up to three years after acute infection compared to non-infected controls, as expressed in ‘disability-adjusted life years’ (DALY) [36, 37].

## 8. Conclusions

Even after the end of the SARS-CoV-2 pandemic, with high burden due to acute infections, long COVID implies longterm and difficult-to-assess consequences for the health of the population and challenges for the healthcare system. On the basis of population-based, controlled studies, approximately 10 to 15 % of adults show long COVID symptoms following SARS-CoV-2 infection, with decreasing risk over time. Nevertheless, given the high number of (re)infections and the cumulative increase in cases with prolonged or chronic symptoms, it can still be assumed that long COVID symptoms are common in the population. However, the full extent of long COVID and its associated public health consequences cannot yet be estimated, given that recent scientific evidence suggests a direct link between SARS-CoV-2 infections and the higher incidence of chronic, non-communicable diseases.

Further research is needed, as the clinical presentation, pathophysiological mechanisms, and preventive and therapeutic options for long COVID still remain poorly understood. This applies in particular to those who are severely affected with long-term or chronic health problems (such as ME/CFS) and their impact on quality of life, everyday functioning, social participation and the medical care needs of those affected. In order to reliably map long-term recovery and relapse rates for long COVID and identify potential predictors for different subtypes and forms of progression, longitudinal studies are particularly necessary to examine the health status of those affected over a longer period of time.

The possible occurrence of post-acute infection syndromes has already been reported in association with other viral infections. However, the scale of the SARS-CoV-2 pandemic has brought this into focus and emphasised the need for further research into the complex link between acute infectious diseases and chronic long-term health effects – also with regard to pandemic preparedness for future viral outbreaks. To ensure comprehensive healthcare, it is essential to foster intensive collaboration between basic, clinical, and epidemiological research, to expand evidence-based science communication, and to integrate preventive and therapeutic measures for long COVID and other post-acute infection syndromes into existing care structures.

## References

[1] Callard F Perego E. How and why patients made Long Covid. Social Science & Medicine. 2021;268:113426. doi: 10.1016/j.socscimed.2020.113426. 33199035 10.1016/j.socscimed.2020.113426 PMC7539940

[2] World Health Organization (WHO). A clinical case definition of post COVID-19 condition by a Delphi consensus, 6 October 2021. 2021 [cited 14.06.2025]. Available from: https://www.who.int/publications/i/item/WHO-2019-nCoV-Post_COVID-19_condition-Clinical_case_definition-2021.1.

[3] Centers for Disease Control and Prevention (CDC). New ICD-10-CM code for post-COVID conditions, following the 2019 novel coronavirus (COVID-19). 2021 [cited 22.06.2025]. Available from: https://www.cdc.gov/nchs/data/icd/announcement-new-icd-code-for-post-covidcondition-april-2022-final.pdf.

[4] Bundesinstitut für Arzneimittel und Medizinprodukte (BfArM). ICD-10-GM Version 2021. Kapitel XXII Schlüsselnummern für besondere Zwecke (U00-U99). 2021 [cited 10.07.2025]. Available from: https://klassifikationen.bfarm.de/icd-10-gm/kode-suche/htmlgm2021/block-u00-u49.htm.

[5] Al-Aly Z Davis H McCorkell L Soares L Wulf-Hanson S Iwasaki A. Long COVID science, research and policy. Nat Med. 2024;30(8): 2148–64. doi: 10.1038/s41591-024-03173-6. 39122965 10.1038/s41591-024-03173-6

[6] Peluso MJ Deeks SG. Mechanisms of long COVID and the path to-ward therapeutics. Cell. 2024;187(20):5500–29. doi: 10.1016/j. cell.2024.07.054. 39326415 10.1016/j.cell.2024.07.054 PMC11455603

[7] Franco JVA Garegnani LI Metzendorf MI Heldt K Mumm R Scheidt-Nave C. Post-covid-19 conditions in adults: systematic review and meta-analysis of health outcomes in controlled studies. BMJ Med. 2024;3(1):e000723. doi: 10.1136/bmjmed-2023-000723. 10.1136/bmjmed-2023-000723 PMC10826558 38293681

[8] Skevaki C Moschopoulos CD Fragkou PC Grote K Schieffer E Schieffer B. Long COVID: Pathophysiology, current concepts, and future directions. J Allergy Clin Immunol. 2025;155(4):1059–70. doi: 10.1016/j.jaci.2024.12.1074. 39724975 10.1016/j.jaci.2024.12.1074

[9] LitCovid. Long COVID Post-COVID and Sequelae – Monthly publications 2025 [cited 10.07.2025]. Available from: https://www.ncbi.nlm.nih.gov/research/coronavirus/docsum?filters=e_condition.LongCovid.

[10] Gutzeit J Weiß M Nürnberger C Lemhöfer C Appel KS Pracht E. Definitions and symptoms of the post-COVID syndrome: an updated systematic umbrella review. Eur Arch Psychiatry Clin Neurosci. 2025;275(1):129–40. doi: 10.1007/s00406-024-01868-y. 39052056 10.1007/s00406-024-01868-y PMC11799012

[11] Organisation for Economic Co-operation and Development (OECD). The prevalence and impact of Long COVID in the primary care population: Findings from the OECD PaRIS survey. 2025 [cited 23.06.2025]. Available from: https://www.oecd.org/en/publications/the-prevalence-and-impact-of-long-covid-in-the-primary-care-population_119b0e8f-en.html.

[12] National Institute for Health and Care Excellence (NICE). COVID-19 rapid guideline: managing the long-term effects of COVID-19 (NICE Guideline NG188). London: National Institute for Health and Care Excellence (UK); 2020 [cited 23.06.2025]. Available from: https://www.nice.org.uk/guidance/ng188.

[13] Toepfner N Brinkmann F Augustin S Stojanov S Behrends U. Long COVID in pediatrics-epidemiology, diagnosis, and management. Eur J Pediatr. 2024;183(4):1543–53. doi: 10.1007/s00431-023-05360-y. 38279014 10.1007/s00431-023-05360-y PMC11001657

[14] Groff D Sun A Ssentongo AE Ba DM Parsons N Poudel GR. Short-term and Long-term Rates of Postacute Sequelae of SARS-CoV-2 Infection: A Systematic Review. JAMA Netw Open. 2021;4(10): e2128568. doi: 10.1001/jamanetworkopen.2021.28568. 34643720 10.1001/jamanetworkopen.2021.28568 PMC8515212

[15] Hallek M Adorjan K Behrends U Ertl G Suttorp N Lehmann C. Post-COVID Syndrome. Dtsch Arztebl Int. 2023;120(4):48–55. doi: 10.3238/arztebl.m2022.0409. 36633452 10.3238/arztebl.m2022.0409 PMC10060997

[16] Koczulla A Ankermann T Behrends U Berlit P Berner R Böing S. S1-Leitlinie “Long/Post-COVID” – Living guideline. 2024 [cited 18.07.2025]. Available from: https://register.awmf.org/de/leitlinien/detail/020-027.

[17] Franco JVA Garegnani LI Oltra GV Metzendorf MI Trivisonno LF Sgarbossa N. Long-Term Health Symptoms and Sequelae Following SARS-CoV-2 Infection: An Evidence Map. Int J Environ Res Public Health. 2022;19(16). doi: 10.3390/ijerph19169915. 10.3390/ijerph19169915 PMC9408764 36011562

[18] World Health Organization (WHO). A clinical case definition for post COVID-19 condition in children and adolescents by expert consensus, 16 February 2023. 2023 [cited 18.07.2024]. Available from: https://www.who.int/publications/i/item/WHO-2019-nCoV-Post-COVID-19-condition-CA-Clinical-case-definition-2023-1.

[19] National Academies of Sciences, Engineering and Medicine. A Long COVID Definition: A Chronic, Systemic Disease State with Profound Consequences. Washington, DC: The National Academies Press; 2024 [cited 04.06.2025]. Available from: https://nap.nationalacademies.org/catalog/27768/a-long-covid-definition-a-chronic-systemicdisease-state-with. 39110819

[20] Global Burden of Disease Long COVID Collaborators. Estimated Global Proportions of Individuals With Persistent Fatigue, Cognitive, and Respiratory Symptom Clusters Following Symptomatic COVID-19 in 2020 and 2021. JAMA. 2022;328(16):1604–15. doi: 10.1001/jama.2022.18931. 36215063 10.1001/jama.2022.18931 PMC9552043

[21] Munblit D Nicholson T Akrami A Apfelbacher C Chen J De Groote W. A core outcome set for post-COVID-19 condition in adults for use in clinical practice and research: an international Delphi consensus study. Lancet Respir Med. 2022;10(7):715–24. doi: 10.1016/s2213-2600(22)00169-2. 35714658 10.1016/S2213-2600(22)00169-2 PMC9197249

[22] Taher MK Salzman T Banal A Morissette K Domingo FR Cheung AM. Global prevalence of post-COVID-19 condition: a systematic review and meta-analysis of prospective evidence. Health Promot Chronic Dis Prev Can. 2025;45(3):112–38. doi: 10.24095/hpcdp.45.3.02. 40073162 10.24095/hpcdp.45.3.02 PMC12039764

[23] Geng LN Erlandson KM Hornig M Letts R Selvaggi C Ashktorab H. 2024 Update of the RECOVER-Adult Long COVID Research Index. Jama. 2025;333(8):694–700. doi: 10.1001/jama.2024.24184. 39693079 10.1001/jama.2024.24184 PMC11862971

[24] Xu Z Wang W Zhang D Tam KW Li Y Chan DCC. Excess risks of long COVID symptoms compared with identical symptoms in the general population: A systematic review and meta-analysis of studies with control groups. J Glob Health. 2024;14:05022. doi: 10.7189/jogh.14.05022. 39129538 10.7189/jogh.14.05022 PMC11317913

[25] O’Mahoney LL Routen A Gillies C Jenkins SA Almaqhawi A Ayoubkhani D. The risk of Long Covid symptoms: a systematic review and meta-analysis of controlled studies. Nature Communications. 2025;16(1):4249. doi: 10.1038/s41467-025-59012-w. 10.1038/s41467-025-59012-w PMC12059169 40335476

[26] Deutsche Gesellschaft für ME/CFS e.V. Post-Exertionelle Malaise. 2025 [cited 16.06.2025]. Available from: https://www.mecfs.de/was-ist-me-cfs/pem/.

[27] Deutsche Gesellschaft für ME/CFS e.V. Was ist ME/CFS? 2025 [cited 31.03.2025]. Available from: https://www.mecfs.de/was-ist-me-cfs/.

[28] Renz-Polster H Scheibenbogen C. [Post-COVID syndrome with fatigue and exercise intolerance: myalgic encephalomyelitis/chronic fatigue syndrome]. Inn Med (Heidelb). 2022;63(8):830–9. doi: 10.1007/s00108-022-01369-x. 35925074 10.1007/s00108-022-01369-x PMC9281337

[29] Wong TL Weitzer DJ. Long COVID and Myalgic Encephalomyelitis/Chronic Fatigue Syndrome (ME/CFS)-A Systemic Review and Comparison of Clinical Presentation and Symptomatology. Medicina (Kaunas). 2021;57(5). doi: 10.3390/medicina57050418. 10.3390/medicina57050418 PMC8145228 33925784

[30] Kedor C Freitag H Meyer-Arndt L Wittke K Hanitsch LG Zoller T. A prospective observational study of post-COVID-19 chronic fatigue syndrome following the first pandemic wave in Germany and biomarkers associated with symptom severity. Nature Communications. 2022;13(1):5104. doi: 10.1038/s41467-022-32507-6. 10.1038/s41467-022-32507-6 PMC9426365 36042189

[31] Kedor C Freitag H Meyer-Arndt L Wittke K Hanitsch LG Zoller T. Author Correction: A prospective observational study of post-COVID-19 chronic fatigue syndrome following the first pandemic wave in Germany and biomarkers associated with symptom severity. Nat Commun. 2022;13(1):6009. doi: 10.1038/s41467-022-33784-x. 36224219 10.1038/s41467-022-33784-x PMC9554845

[32] Roessler M Tesch F Batram M Jacob J Loser F Weidinger O. Post-COVID-19-associated morbidity in children, adolescents, and adults: A matched cohort study including more than 157,000 individuals with COVID-19 in Germany. PLOS Medicine. 2022;19(11): e1004122. doi: 10.1371/journal.pmed.1004122. 36355754 10.1371/journal.pmed.1004122 PMC9648706

[33] Dehlia A Guthridge MA. The persistence of myalgic encephalomyelitis/chronic fatigue syndrome (ME/CFS) after SARS-CoV-2 infection: A systematic review and meta-analysis. J Infect. 2024;89(6):106297. doi: 10.1016/j.jinf.2024.106297. 39353473 10.1016/j.jinf.2024.106297

[34] Al-Aly Z Xie Y Bowe B. High-dimensional characterization of postacute sequelae of COVID-19. Nature. 2021;594(7862):259–64. doi: 10.1038/s41586-021-03553-9. 33887749 10.1038/s41586-021-03553-9

[35] Taquet M Sillett R Zhu L Mendel J Camplisson I Dercon Q. Neurological and psychiatric risk trajectories after SARS-CoV-2 infection: an analysis of 2-year retrospective cohort studies including 1 284 437 patients. Lancet Psychiatry. 2022;9(10):815–27. doi: 10.1016/s2215-0366(22)00260-7. 35987197 10.1016/S2215-0366(22)00260-7 PMC9385200

[36] Bowe B Xie Y Al-Aly Z. Postacute sequelae of COVID-19 at 2 years. Nature Medicine. 2023;29(9):2347–57. doi: 10.1038/s41591-023-02521-2. 10.1038/s41591-023-02521-2 PMC10504070 37605079

[37] Cai M Xie Y Topol EJ Al-Aly Z. Three-year outcomes of post-acute sequelae of COVID-19. Nat Med. 2024;30(6):1564–73. doi: 10.1038/s41591-024-02987-8. 38816608 10.1038/s41591-024-02987-8 PMC11186764

[38] Heidemann C Sarganas G Du Y Gaertner B Poethko-Müller C Cohrdes C. Long-term health consequences among individuals with SARS-CoV-2 infection compared to individuals without infection: results of the population-based cohort study CoMoLo Follow-up. BMC Public Health. 2023;23(1):1587. doi: 10.1186/s12889-023-16524-8. 37605232 10.1186/s12889-023-16524-8 PMC10440884

[39] Tesch F Ehm F Vivirito A Wende D Batram M Loser F. Incident autoimmune diseases in association with SARS-CoV-2 infection: a matched cohort study. Clin Rheumatol. 2023;24(1):1126. doi: 10.1007/s10067-023-06670-0. 10.1007/s10067-023-06670-0 PMC10497688 37335408

[40] Schmitt J Ehm F Vivirito A Wende D Batram M Loser F. Large cohort study shows increased risk of developing atopic dermatitis after COVID-19 disease. Allergy. 2024;79(1):232–4. doi: 10.1111/all.15827. 37469301 10.1111/all.15827

[41] Choutka J Jansari V Hornig M Iwasaki A. Unexplained post-acute infection syndromes. Nat Med. 2022;28(5):911–23. doi: 10.1038/s41591022-01810-6. 35585196 10.1038/s41591-022-01810-6

[42] Xie Y Choi T Al-Aly Z. Long-term outcomes following hospital admission for COVID-19 versus seasonal influenza: a cohort study. The Lancet Infectious Diseases. 2024;24(3):239–55. doi: 10.1016/S14733099(23)00684-9. 38104583 10.1016/S1473-3099(23)00684-9

[43] Liu TH Huang PY Wu JY Chuang MH Hsu WH Tsai YW. Comparison of post-acute sequelae following hospitalization for COVID-19 and influenza. BMC Medicine. 2023;21(1):480. doi: 10.1186/s12916-023-03200-2. 38049876 10.1186/s12916-023-03200-2 PMC10696681

[44] Fung KW Baye F Baik SH Zheng Z McDonald CJ. Prevalence and characteristics of long COVID in elderly patients: An observational cohort study of over 2 million adults in the US. PLoS Med. 2023;20(4): e1004194. doi: 10.1371/journal.pmed.1004194. 37068113 10.1371/journal.pmed.1004194 PMC10150975

[45] Taquet M Dercon Q Luciano S Geddes JR Husain M Harrison PJ. Incidence, co-occurrence, and evolution of long-COVID features: A 6-month retrospective cohort study of 273,618 survivors of COVID-19. PLOS Medicine. 2021;18(9):e1003773. doi: 10.1371/journal.pmed. 1003773. 34582441 10.1371/journal.pmed.1003773 PMC8478214

[46] Taquet M Geddes JR Husain M Luciano S Harrison PJ. 6-month neurological and psychiatric outcomes in 236 379 survivors of COVID-19: a retrospective cohort study using electronic health records. The Lancet Psychiatry. 2021;8(5):416–27. doi: 10.1016/S22150366(21)00084-5. 33836148 10.1016/S2215-0366(21)00084-5 PMC8023694

[47] Tesch F Ehm F Loser F Bechmann L Vivirito A Wende D. Post-viral symptoms and conditions are more frequent in COVID-19 than influenza, but not more persistent. BMC Infect Dis. 2024;24(1): 1126. doi: 10.1186/s12879-024-10059-y. 39385128 10.1186/s12879-024-10059-y PMC11465902

[48] Oseran AS Song Y Xu J Dahabreh IJ Wadhera RK de Lemos JA. Long term risk of death and readmission after hospital admission with covid-19 among older adults: retrospective cohort study. Bmj. 2023;382:e076222. doi: 10.1136/bmj-2023-076222. 37558240 10.1136/bmj-2023-076222 PMC10475839

[49] Bullock A Dalton AF Stockwell MS McLaren SH Sano E Nguyen HQ. Ongoing Symptoms After Acute SARS-CoV-2 or Influenza Infection in a Case-Ascertained Household Transmission Study: 7 US Sites, 2021-2023. Clin Infect Dis. 2025;80(5):1032–44. doi: 10.1093/cid/ciaf026. 40036243 10.1093/cid/ciaf026 PMC12159730

[50] Carr CR Gentile NL Bertolli J Szewczyk W Lin JS Unger ER. Comparison of long COVID, recovered COVID, and non-COVID Post-Acute Infection Syndromes over three years. PLoS One. 2025;20(5): e0323104. doi: 10.1371/journal.pone.0323104. 40393039 10.1371/journal.pone.0323104 PMC12092011

[51] Wee LE Ho RWL Lim JT Chiew CJ Lye DCB Tan KB. Long-term multi-systemic sequelae post-hospitalization for Omicron COVID-19 versus influenza: a retrospective cohort study. Int J Infect Dis. 2025: 107946. doi: 10.1016/j.ijid.2025.107946. 40499677 10.1016/j.ijid.2025.107946

[52] Hua MJ Butera G Akinyemi O Porterfield D. Biases and limitations in observational studies of Long COVID prevalence and risk factors: A rapid systematic umbrella review. PLoS One. 2024;19(5):e0302408. doi: 10.1371/journal.pone.0302408. 38696415 10.1371/journal.pone.0302408 PMC11065234

[53] Woodrow M Carey C Ziauddeen N Thomas R Akrami A Lutje V. Systematic Review of the Prevalence of Long COVID. Open Forum Infect Dis. 2023;10(7):ofad233. doi: 10.1093/ofid/ofad233. 37404951 10.1093/ofid/ofad233 PMC10316694

[54] Nittas V Gao M West EA Ballouz T Menges D Wulf Hanson S. Long COVID Through a Public Health Lens: An Umbrella Review. Public Health Rev. 2022;43:1604501. doi: 10.3389/phrs.2022.1604501. 35359614 10.3389/phrs.2022.1604501 PMC8963488

[55] Ford ND Slaughter D Edwards D Dalton A Perrine C Vahratian A Long COVID and Significant Activity Limitation Among Adults, by Age – United States, June 1-13,. 2022, to June 7-19, 2023. MMWR Morb Mortal Wkly Rep. 2023;72(32):866–70. doi: 10.15585/mmwr. mm7232a3. 37561665 10.15585/mmwr.mm7232a3 PMC10415000

[56] Office of National Statistics (ONS). Prevalence of ongoing symptoms following coronavirus (COVID-19) infection in the UK: 30 March 2023. 2023 [cited 15.07.2025]. Available from: https://www.ons.gov.uk/peoplepopulationandcommunity/healthandsocialcare/conditionsanddiseases/bulletins/prevalenceofongoingsymptomsfollowingcoronaviruscovid19infectionintheuk/30march2023.

[57] Thompson EJ Williams DM Walker AJ Mitchell RE Niedzwiedz CL Yang TC. Long COVID burden and risk factors in 10 UK longitudinal studies and electronic health records. Nat Commun. 2022;13(1):3528. doi: 10.1038/s41467-022-30836-0. 35764621 10.1038/s41467-022-30836-0 PMC9240035

[58] Coste J Delpierre C Richard JB Alleaume C Gallay A Tebeka S. Prevalence of long COVID in the general adult population according to different definitions and sociodemographic and infection characteristics. A nationwide random sampling survey in France in autumn 2022. Clin Microbiol Infect. 2024;30(7):924–9. doi: 10.1016/j.cmi.2024.03.020. 38527615 10.1016/j.cmi.2024.03.020

[59] Peter RS Nieters A Kräusslich HG Brockmann SO Göpel S Kindle G. Post-acute sequelae of covid-19 six to 12 months after infection: population based study. BMJ. 2022;379:e071050. doi: 10.1136/bmj-2022-071050. 36229057 10.1136/bmj-2022-071050 PMC9557001

[60] Hastie CE Lowe DJ McAuley A Mills NL Winter AJ Black C. True prevalence of long-COVID in a nationwide, population cohort study. Nat Commun. 2023;14(1):7892. doi: 10.1038/s41467-023-43661-w. 38036541 10.1038/s41467-023-43661-w PMC10689486

[61] Ballering AV van Zon SKR olde Hartman TC Rosmalen JGM. Persistence of somatic symptoms after COVID-19 in the Netherlands: an observational cohort study. The Lancet. 2022;400(10350):452–61. doi: 10.1016/S0140-6736(22)01214-4. 10.1016/S0140-6736(22)01214-4 PMC9352274 35934007

[62] Fernández-de-Las-Peñas C Notarte KI Peligro PJ Velasco JV Ocampo MJ Henry BM. Long-COVID Symptoms in Individuals Infected with Different SARS-CoV-2 Variants of Concern: A Systematic Review of the Literature. Viruses. 2022;14(12). doi: 10.3390/v14122629. 10.3390/v14122629 PMC9785120 36560633

[63] Beale S Yavlinsky A Fong WLE Nguyen VG Kovar J Vos T. Long-term outcomes of SARS-CoV-2 variants and other respiratory infections: evidence from the Virus Watch prospective cohort in England. Epidemiol Infect. 2024;152:e77. doi: 10.1017/s0950268824 000748. 38724258 10.1017/S0950268824000748 PMC11106725

[64] Hedberg P Nauclér P. Post-COVID-19 Condition After SARS-CoV-2 Infections During the Omicron Surge vs the Delta, Alpha, and Wild Type Periods in Stockholm, Sweden. J Infect Dis. 2024;229(1):133–6. doi: 10.1093/infdis/jiad382. 37665981 10.1093/infdis/jiad382 PMC10786247

[65] Swift MD Breeher LE Dierkhising R Hickman J Johnson MG Roellinger DL. Association of COVID-19 Vaccination With Risk of Medically Attended Postacute Sequelae of COVID-19 During the Ancestral, Alpha, Delta, and Omicron Variant Eras. Open Forum Infect Dis. 2024;11(9):ofae495. doi: 10.1093/ofid/ofae495. 39290777 10.1093/ofid/ofae495 PMC11406745

[66] Thaweethai T Jolley SE Karlson EW Levitan EB Levy B McComsey GA. Development of a Definition of Postacute Sequelae of SARS-CoV-2 Infection. Jama. 2023;329(22):1934–46. doi: 10.1001/jama.2023.8823. 37278994 10.1001/jama.2023.8823 PMC10214179

[67] Caspersen IH Skodvin SN Blix K Robertson AH Laake I Feiring B. Post-COVID symptoms after SARS-CoV-2 omicron infection and the effect of booster vaccination: A population-based cohort study. Vaccine. 2025;47:126664. doi: 10.1016/j.vaccine.2024.126664. 39787799 10.1016/j.vaccine.2024.126664

[68] Mikolajczyk R Diexer S Klee B Pfrommer L Purschke O Fricke J. Likelihood of Post-COVID Condition in people with hybrid immunity; data from the German National Cohort (NAKO). J Infect. 2024; 89(2):106206. doi: 10.1016/j.jinf.2024.106206. 38897239 10.1016/j.jinf.2024.106206

[69] Xie Y Choi T Al-Aly Z. Postacute Sequelae of SARS-CoV-2 Infection in the Pre-Delta, Delta, and Omicron Eras. N Engl J Med. 2024;391(6): 515–25. doi: 10.1056/NEJMoa2403211. 39018527 10.1056/NEJMoa2403211 PMC11687648

[70] Hori M Hayama-Terada M Kitamura A Hosozawa M Muto Y Iba A. Risk factors for post-coronavirus disease condition in the Alpha-, Delta-, and Omicron-dominant waves among adults in Japan: A population-based matched case-control study. J Med Virol. 2024; 96(9):e29928. doi: 10.1002/jmv.29928. 39311094 10.1002/jmv.29928

[71] Valdivieso-Martinez B Lopez-Sanchez V Sauri I Diaz J Calderon JM Gas-Lopez ME. Impact of Long SARS-CoV-2 Omicron Infection on the Health Care Burden: Comparative Case-Control Study Between Omicron and Pre-Omicron Waves. JMIR Public Health Surveill. 2024;10:e53580. doi: 10.2196/53580. 39226091 10.2196/53580 PMC11408891

[72] Ballouz T Menges D Kaufmann M Amati R Frei A von Wyl V. Post COVID-19 condition after Wildtype, Delta, and Omicron SARS-CoV-2 infection and prior vaccination: Pooled analysis of two population-based cohorts. PLoS One. 2023;18(2):e0281429. doi: 10.1371/journal.pone.0281429. 36812215 10.1371/journal.pone.0281429 PMC9946205

[73] Luo D Mei B Wang P Li X Chen X Wei G. Prevalence and risk factors for persistent symptoms after COVID-19: a systematic review and meta-analysis. Clin Microbiol Infect. 2024;30(3):328–35. doi: 10.1016/j.cmi.2023.10.016. 37866679 10.1016/j.cmi.2023.10.016

[74] Muley A Mitra S Bhaliya B Soni S Joshi A. A Systematic Review and Meta-analysis to Identify Risk Factors for Developing Long COVID-19. J Assoc Physicians India. 2024;72(5):68–74. doi: 10.59556/japi. 72.0528. 10.59556/japi.72.0528 38881113

[75] Tsampasian V Elghazaly H Chattopadhyay R Debski M Naing TKP Garg P. Risk Factors Associated With Post-COVID-19 Condition: A Systematic Review and Meta-analysis. JAMA Intern Med. 2023; 183(6):566–80. doi: 10.1001/jamainternmed.2023.0750. 36951832 10.1001/jamainternmed.2023.0750 PMC10037203

[76] Terry P Heidel RE Wilson AQ Dhand R. Risk of long covid in patients with pre-existing chronic respiratory diseases: a systematic review and meta-analysis. BMJ Open Respir Res. 2025;12(1). doi: 10.1136/bmjresp-2024-002528. 10.1136/bmjresp-2024-002528 PMC11784193 39884720

[77] Sha‘ari NI Ismail A Abdul Aziz AF Suddin LS Azzeri A Sk Abd Razak R. Cardiovascular diseases as risk factors of post-COVID syndrome: a systematic review. BMC Public Health. 2024;24(1):1846. doi: 10.1186/s12889-024-19300-4. 38987743 10.1186/s12889-024-19300-4 PMC11238467

[78] Wang Y Su B Alcalde-Herraiz M Barclay NL Tian Y Li C. Modifiable lifestyle factors and the risk of post-COVID-19 multisystem sequelae, hospitalization, and death. Nat Commun. 2024;15(1):6363. doi: 10.1038/s41467-024-50495-7. 39075060 10.1038/s41467-024-50495-7 PMC11286928

[79] Gorenshtein A Leibovitch L Liba T Stern S Stern Y. Gender Disparities in Neurological Symptoms of Long-COVID: A systematic review and meta-analysis. Neuroepidemiology. 2024;59(4):426–40. doi: 10.1159/000540919. 39159607 10.1159/000540919 PMC12324708

[80] D‘Onofrio V Sékaly RP. The immune-endocrine interplay in sex differential responses to viral infection and COVID-19. Trends Immunol. 2024;45(12):943–58. doi: 10.1016/j.it.2024.10.004. 39562265 10.1016/j.it.2024.10.004

[81] Silva J Iwasaki A. Sex differences in postacute infection syndromes. Sci Transl Med. 2024;16(773):eado2102. doi: 10.1126/scitranslmed. ado2102. 39536120 10.1126/scitranslmed.ado2102 PMC12805797

[82] Al-Aly Z Topol E. Solving the puzzle of Long Covid. Science. 2024; 383(6685):830–2. doi: 10.1126/science.adl0867. 38386747 10.1126/science.adl0867

[83] Lammers N Beese F Hoebel J Poethko-Müller C Wachtler B. Social Inequalities in Long-Term Health Effects After COVID-19-A Scoping Review. Int J Public Health. 2024;69:1606739. doi: 10.3389/ijph.2024.1606739. 38384747 10.3389/ijph.2024.1606739 PMC10878999

[84] Subramanian A Nirantharakumar K Hughes S Myles P Williams T Gokhale KM. Symptoms and risk factors for long COVID in non-hospitalized adults. Nat Med. 2022;28(8):1706–14. doi: 10.1038/s41591-022-01909-w. 35879616 10.1038/s41591-022-01909-w PMC9388369

[85] Lukkahatai N Rodney T Ling C Daniel B Han HR. Long COVID in the context of social determinants of health. Front Public Health. 2023;11:1098443. doi: 10.3389/fpubh.2023.1098443. 37056649 10.3389/fpubh.2023.1098443 PMC10088562

[86] Chow KN Tsang YW Chan YH Telaga SA Ng LYA Chung CM. The effect of pre-COVID and post-COVID vaccination on long COVID: a systematic review and meta-analysis. J Infect. 2024:106358. doi: 10.1016/j.jinf.2024.106358. 39580033 10.1016/j.jinf.2024.106358

[87] European Centre for Disease Prevention and Control (ECDC). Does COVID-19 vaccination reduce the risk and duration of post-COVID-19 condition? Rapid systematic literature review. 2025 [cited 14.06.2025]. Available from: https://www.ecdc.europa.eu/sites/default/files/documents/does-COVID-19-vaccination-reduce-risk-duration-post-COVID-19-condition-March-2025.pdf.

[88] Sterian M Naganathan T Corrin T Waddell L. Evidence on the associations and safety of COVID-19 vaccination and post COVID-19 condition: an updated living systematic review. Epidemiol Infect. 2025;153: e62. doi: 10.1017/s0950268825000378. 40159916 10.1017/S0950268825000378 PMC12038765

[89] Choi YJ Seo YB Seo JW Lee J Nham E Seong H. Effectiveness of Antiviral Therapy on Long COVID: A Systematic Review and Meta-Analysis. J Clin Med. 2023;12(23). doi: 10.3390/jcm12237375. 10.3390/jcm12237375 PMC10707593 38068427

[90] Sun G Lin K Ai J Zhang W. The efficacy of antivirals, corticosteroids, and monoclonal antibodies as acute COVID-19 treatments in reducing the incidence of long COVID: a systematic review and meta-analysis. Clin Microbiol Infect. 2024;30(12):1505–13. doi: 10.1016/j.cmi.2024.07.006. 39002665 10.1016/j.cmi.2024.07.006

[91] Bowe B Xie Y Al-Aly Z. Acute and postacute sequelae associated with SARS-CoV-2 reinfection. Nature Medicine. 2022;28(11):2398–405. doi: 10.1038/s41591-022-02051-3. 10.1038/s41591-022-02051-3 PMC9671810 36357676

[92] Bosworth ML Shenhuy B Walker AS Nafilyan V Alwan NA O‘Hara ME. Risk of New-Onset Long COVID Following Reinfection With Severe Acute Respiratory Syndrome Coronavirus 2: A Community-Based Cohort Study. Open Forum Infect Dis. 2023;10(11):ofad493. doi: 10.1093/ofid/ofad493. 37953820 10.1093/ofid/ofad493 PMC10633780

[93] Peter RS Nieters A Göpel S Merle U Steinacker JM Deibert P. Persistent symptoms and clinical findings in adults with post-acute sequelae of COVID-19/post-COVID-19 syndrome in the second year after acute infection: A population-based, nested case-control study. PLoS Med. 2025;22(1):e1004511. doi: 10.1371/journal.pmed.1004511. 39847575 10.1371/journal.pmed.1004511 PMC12005676

[94] Pfrommer LR Diexer S Klee B Massag J Gottschick C Purschke O. Post-COVID recovery is faster after an infection with the SARS-CoV-2 Omicron variant: a population-based cohort study. Infection. 2025;53(2):657–65. doi: 10.1007/s15010-024-02438-z. 39556163 10.1007/s15010-024-02438-z PMC11971134

[95] Rahmati M Udeh R Kang J Dolja-Gore X McEvoy M Kazemi A. Long-Term Sequelae of COVID-19: A Systematic Review and Meta-Analysis of Symptoms 3 Years Post-SARS-CoV-2 Infection. Journal of Medical Virology. 2025;97(6):e70429. doi: 10.1002/jmv.70429. 40476637 10.1002/jmv.70429 PMC12143191

[96] Huang Q Jia M Sun Y Jiang B Cui D Feng L. One-Year Temporal Changes in Long COVID Prevalence and Characteristics: A Systematic Review and Meta-Analysis. Value Health. 2023;26(6):934–42. doi: 10.1016/j.jval.2022.11.011. 36436792 10.1016/j.jval.2022.11.011

[97] Hartung TJ Bahmer T Chaplinskaya-Sobol I Deckert J Endres M Franzpotter K. Predictors of non-recovery from fatigue and cognitive deficits after COVID-19: a prospective, longitudinal, population-based study. EClinicalMedicine. 2024;69:102456. doi: 10.1016/j.eclinm.2024.102456. 38333368 10.1016/j.eclinm.2024.102456 PMC10847699

[98] Rawal G Yadav S Kumar R. Post-intensive Care Syndrome: an Overview. J Transl Int Med. 2017;5(2):90–2. doi: 10.1515/jtim-2016-0016. 28721340 10.1515/jtim-2016-0016 PMC5506407

[99] Bundesinstitut für Arzneimittel und Medizinprodukte (BfArM). Expertengruppe Long COVID Off-Label-Use. [cited 22.06.2025]. Available from: https://www.bfarm.de/DE/Arzneimittel/Zulassung/Zulas-sungsrelevante-Themen/Expertengruppe-Long-COVID-Off-Label-Use/_node.html.

[100] Expertengruppe Long COVID Off-Label-Use. Long COVID – Arzneimittel: Maßnahmen zur Verbesserung der Versorgung von Long COVID-Erkrankten. [cited 22.05.2025]. Available from: https://www.bfarm.de/SharedDocs/Downloads/DE/Arzneimittel/Zulassung/Zul-RelThemen/LongCOVID/therapie-kompass.html.

[101] World Health Organization (WHO). Long COVID Rehabilitation Guidelines 2022 [cited 22.06.2025]. Available from: https://longcovid.physio/our-work/who-long-covid-rehab-guidelines.

[102] Gloeckl R Leitl D Schneeberger T Jarosch I Koczulla AR. Rehabilitative interventions in patients with persistent post COVID-19 symptoms-a review of recent advances and future perspectives. Eur Arch Psychiatry Clin Neurosci. 2024;274(8):1819–28. doi: 10.1007/s00406-023-01631-9. 37326700 10.1007/s00406-023-01631-9 PMC11579067

[103] Schulz M Mangiapane S Scherer M Karagiannidis C Czihal T. Post-Acute Sequelae of SARS-CoV-2 Infection. Dtsch Arztebl Int. 2022;119(10):177–8. doi: 10.3238/arztebl.m2022.0134. 35583040 10.3238/arztebl.m2022.0134 PMC9215269

[104] Platz T Berlit P Dohle C Fickenscher H Guha M Köllner V. S2k-Guideline SARS-CoV-2, COVID-19 and (early) rehabilitation - a consensus-based guideline for Germany. GMS Hyg Infect Control. 2023;18:Doc12. doi: 10.3205/dgkh000438. 37261059 10.3205/dgkh000438 PMC10227492

[105] Deutsche Rentenversicherung Bund (DRV). Eckpunktepapier für die medizinische Rehabilitation bei Post-COVID-Syndrom. Stand. 2023 [cited 24.06.2025]. Available from: https://www.deutsche-rentenver-sicherung.de/SharedDocs/Downloads/DE/Experten/infos_reha_einrichtungen/eckpunkte-reha-post-covid-syndrom-10-2023.html.

[106] Gemeinsamer Bundesausschuss (G-BA). Long-COVID-Richtlinie. 2024 [cited 15.06.2025]. Available from: https://www.g-ba.de/richtlinien/141/.

[107] Kassenärztliche Bundesvereinigung (KBV). Neue EBM-Leistungen für Patienten mit Verdacht auf Long COVID. 2024 [cited 22.06.2025]. Available from: https://www.kbv.de/html/1150_73128.php.

[108] Kassenärztliche Bundesvereinigung (KBV). Diagnoseliste langfristiger Heilmittelbedarf/Besonderer Verordnungsbedarf/Blankoverordnung. Stand 1. Oktober 2025. 2025 [cited 04.06.2025]. Available from: https://www.kbv.de/documents/praxis/verordnungen/heilmittel/heilmittel-diagnoseliste.pdf.

[109] Heuer J Bätzing J Holstiege J Akmatov MK Dammertz L Kohring C. Vertragsärztlich-ambulante Versorgung von COVID-19-Patienten im bundesweiten regionalen Vergleich (Teil 2) – Schwerpunkt 2. und 3. Welle der Pandemie in Deutschland. Zentralinstitut für die kassenärztliche Versorgung in Deutschland (Zi); 2023 [cited 14.06.2025]. Available from: https://www.versorgungsatlas.de/fileadmin/ziva_docs/140/VA-23-04-Ambul-Versorg-Covid-19-Welle2-3_Endversion.pdf.

[110] Monitor-Versorgungsforschung. Über 60 Prozent der Patient:innen mit Post-COVID-19-Diagnose nur in einem Quartal in vertragsärztlicher Behandlung. 2022 [cited 23.06.2025]. Available from: https://www.monitor-versorgungsforschung.de/news/ueber-60-prozent-der-patientinnen-mit-post-covid-19-diagnose-nur-in-einem-quartal-in-vertragsaerztlicher-behandlung/?cookie-state-change=1747997128508.

[111] Zentralinstitut für die kassenärztliche Versorgung (ZI). Deskription von Post-COVID-Patient:innen (Bundesweit, Q3 2024). 2025 [cited 22.06.2025]. Available from: https://www.zi.de/fileadmin/Downloads/Themen/Versorgungsanalysen/Post_COVID/PostCOVID_20243_Bun-desweit_20250404.pdf.

[112] Wei Y Horne EM Knight R Cezard G Walker AJ Fisher L. Patient characteristics associated with clinically coded long COVID: an OpenSAFELY study using electronic health records. BJGP Open. 2025;9(4):BJGPO.2024.0140. doi: 10.3399/bjgpo.2024.0140. 40500151 10.3399/BJGPO.2024.0140 PMC12820521

[113] Woldegiorgis M Cadby G Ngeh S Korda RJ Armstrong PK Maticevic J. Long COVID in a highly vaccinated but largely unexposed Australian population following the 2022 SARS-CoV-2 Omicron wave: a cross-sectional survey. Med J Aust. 2024;220(6):323–30. doi: 10.5694/mja2.52256. 38508863 10.5694/mja2.52256

[114] Weigel B Inderyas M Eaton-Fitch N Thapaliya K Marshall-Gradisnik S. Health-related quality of life in Myalgic Encephalomyelitis/Chronic Fatigue Syndrome and Post COVID-19 Condition: a systematic review. J Transl Med. 2025;23(1):318. doi: 10.1186/s12967-025-06131-z. 40075382 10.1186/s12967-025-06131-z PMC11905571

[115] Hossain MM Das J Rahman F Nesa F Hossain P Islam AMK. Living with „long COVID“: A systematic review and meta-synthesis of qualitative evidence. PLoS One. 2023;18(2):e0281884. doi: 10.1371/journal.pone.0281884. 36795701 10.1371/journal.pone.0281884 PMC9934341

[116] Di Fusco M Cappelleri JC Yehoshua A Craig KJT Alvarez MB Allen KE. Associations between symptom-based long COVID clusters and long-term quality of life, work and daily activities among individuals testing positive for SARS-CoV-2 at a national retail pharmacy. J Patient Rep Outcomes. 2024;8(1):122. doi: 10.1186/s41687-024-00797-7. 39436613 10.1186/s41687-024-00797-7 PMC11496399

[117] Lapin B Li Y Englund K Katzan IL. Health-Related Quality of Life for Patients with Post-Acute COVID-19 Syndrome: Identification of Symptom Clusters and Predictors of Long-Term Outcomes. J Gen Intern Med. 2024;39(8):1301–9. doi: 10.1007/s11606-024-08688-9. 38424349 10.1007/s11606-024-08688-9 PMC11169186

[118] Gonzalez AE Suzuki E. The impacts of long COVID across OECD countries. 2024 [cited 12.06.2025]. Available from: https://www.oecd.org/content/dam/oecd/en/publications/reports/2024/06/the-im-pacts-of-long-covid-across-oecd-countries_f662b21c/8bd08383-en.pdf.

[119] Ottiger M Poppele I Sperling N Schlesinger T Muller K. Work ability and return-to-work of patients with post-COVID-19: a systematic review and meta-analysis. BMC Public Health. 2024;24(1):1811. doi: 10.1186/s12889-024-19328-6. 38973011 10.1186/s12889-024-19328-6 PMC11229229

[120] Muller K Poppele I Ottiger M Wastlhuber A Weber RC Stegbauer M. Long-term course and factors influencing work ability and return to work in post-COVID patients 12 months after inpatient rehabilitation. J Occup Med Toxicol. 2024;19(1):43. doi: 10.1186/s12995-024-00443-4. 39487519 10.1186/s12995-024-00443-4 PMC11529184

[121] Wissenschaftliches Institut der AOK (WIdO). Post-Covid und Long-Covid: Sinkende Zahl von Krankschreibungen, aber weiterhin lange berufliche Fehlzeiten der Betroffenen. 2024 [cited 10.07.2025]. Available from: https://www.aok.de/pp/bv/pm/post-covid-und-long-covid/.

[122] Deutsche Gesetzliche Unfallversicherung (DGUV). Berufskrankheiten und Arbeitsunfälle im Zusammenhang mit Covid-19. 2025 [cited 10.07.2025]. Available from: https://www.dguv.de/medien/inhalt/mediencenter/hintergrund/covid/dguv_zahlencovid.pdf.

[123] Deutsche Rentenversicherung Bund (DRV). Statistik der Deutschen Rentenversicherung – Rentenversicherung in Zahlen 2024. 2024 [cited 10.07.2025]. Available from: https://www.deutsche-rentenver-sicherung.de/SharedDocs/Downloads/DE/Statistiken-und-Berichte/statistikpublikationen/rv_in_zahlen.html.

[124] Calvo Ramos S Maldonado J. E. Vandeplas A. Vanyolos I. Directorate-General for Economic and Financial Affairs. Long COVID: A Tentative Assessment of Its Impact on Labour Market Participation and Potential Economic Effects in the EU. 2024 [cited 14.06.2025]. Available from: https://economy-finance.ec.europa.eu/system/files/2024-01/eb077_en.pdf.

[125] Gottlieb M Yu H Chen J Spatz ES Gentile NL Geyer RE. Differences in Long COVID severity by duration of illness, symptom evolution, and vaccination: a longitudinal cohort study from the INSPIRE group. Lancet Reg Health Am. 2025;44:101026. Epub 20250214. doi: 10.1016/j.lana.2025.101026. 40040820 10.1016/j.lana.2025.101026 PMC11875141

[126] Lukomska E Kloc K Kowalska M Matjaszek A Joshi K Scholz S. Healthcare Resource Utilization (HCRU) and Direct Medical Costs Associated with Long COVID or Post-COVID-19 Conditions: Findings from a Literature Review. J Mark Access Health Policy. 2025;13(1):7. doi: 10.3390/jmahp13010007. 39990183 10.3390/jmahp13010007 PMC11843940

[127] Bartsch SM Chin KL Strych U John DC Shah TD Bottazzi ME. The Current and Future Burden of Long COVID in the United States (U.S.). J Infect Dis. 2025;231(6):1581–90. doi: 10.1093/infdis/jiaf030. 39842946 10.1093/infdis/jiaf030 PMC12247809

[128] Eligulashvili A Gordon M Lee JS Lee J Mehrotra-Varma S Mehrotra-Varma J. Long-term outcomes of hospitalized patients with SARS-CoV-2/COVID-19 with and without neurological involvement: 3-year follow-up assessment. PLoS Med. 2024;21(4):e1004263. doi: 10.1371/journal.pmed.1004263. 38573873 10.1371/journal.pmed.1004263 PMC10994395

